# A Guide to Native Mass Spectrometry to determine complex interactomes of molecular machines

**DOI:** 10.1111/febs.15281

**Published:** 2020-03-25

**Authors:** Rita Puglisi, Elisabetta Boeri Erba, Annalisa Pastore

**Affiliations:** ^1^ UK Dementia Research Institute at the Wohl Institute of King’s College London UK; ^2^ Univ. Grenoble Alpes CEA CNRS Institut de Biologie Structurale (IBS) Grenoble France

**Keywords:** Isc operon; FeS cluster; native mass spectrometry, proteins, structural biology

## Abstract

Native mass spectrometry is an emerging technique in biology that gives the possibility to study noncovalently bound complexes with high sensitivity and accuracy. It thus allows the characterization of macromolecular assemblies, assessing their mass and stoichiometries and mapping the interacting surfaces. In this review, we discuss the application of native mass spectrometry to dynamic molecular machines based on multiple weak interactions. In the study of these machines, it is crucial to understand which and under which conditions various complexes form at any time point. We focus on the specific example of the iron–sulfur cluster biogenesis machine because this is an archetype of a dynamic machine that requires very specific and demanding experimental conditions, such as anaerobicity and the need of retaining the fold of marginally folded proteins. We describe the advantages, challenges and current limitations of the technique by providing examples from our own experience and suggesting possible future solutions.

## Graphical abstract

Native mass spectrometry (MS) can successfully be applied to study the interactome of iron–sulfur cluster biogenesis. In this review, we discuss how native MS spectra can determine all the species present in solution, their stoichiometry and their interactions.

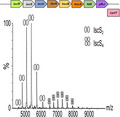

**Abbreviations**
DTT: dithiothreitol
*E. coli*
: *Escherichia coli*
ESI: electrospray ionization
FeS: iron–sulfur
isc: iron–sulfur cluster
MALDI: matrix‐assisted laser desorption/ionization
MS: mass spectrometry
Q‐TOF: nano‐ESI quadrupole time‐of‐flight
Tris: (hydroxymethyl) aminomethane

## Introduction

Mass spectrometry (MS) is a powerful technique that can measure the mass of molecules with high accuracy, sensitivity, resolution and speed [1]. When samples are analysed by MS, they are ionized, separated according to their mass‐to‐charge ratio (*m/z*) and detected. MS was first developed as early as in 1912 [2] and then applied for a long time mostly for analytic purpose to establish or confirm the identity of a compound or a mixture of molecules. Biological applications were allowed only in the late 1980s thanks to the development of soft ionization methods as electrospray ionization (ESI) [3, 4] and matrix‐assisted laser desorption/ionization (MALDI) [5, 6]. ESI in particular led to the possibility, in the ‘90s and around the turning of the millennium, of performing experiments under native conditions (for an early classical review, see Last and Robinson, 1999 [7]). What is now usually referred to as ‘native MS’ thus enables the characterization of macromolecular assemblies without their disassembly [8].

Using native MS, it is in principle possible to determine with high resolution and selectivity the stoichiometry of assembly components [9, 10, 11, 12, 13], map direct interactions between subunits and provide information about the mechanism of formation of macromolecular assemblies [14]. Native MS can also provide information on the dynamic behaviour of complexes and assess the presence of distinct forms that could detect allostery [15, 16]. Another exciting application is the possibility to follow the kinetics of a reaction by incubating mixtures of differently labelled forms of noncovalent complexes (e.g. labelled with ^2^H or ^13^C and ^15^N) [17, 18]. As for all MS applications, native MS has the additional advantage to be undemanding about sample quantities: few picomoles of sample are sufficient for obtaining the mass of even large macromolecular assemblies with an error of a few daltons.

Investigating macromolecular complexes requires customized mass spectrometers, because assemblies with molecular masses above 60 kDa generate ions with *m/z* ratios above 4000 that exceed the detection limit of standard instruments. Around the 2000, major hardware modifications allowed the detection of noncovalent assemblies using nano‐ESI quadrupole time‐of‐flight (Q‐TOF) mass spectrometers ([19, 20]). Besides nano‐ESI‐Q‐TOF instruments, Fourier transform (FT)‐based mass spectrometers have also successfully been used to study noncovalent interactions. In particular, Orbitrap instruments, which were introduced in the year 2000 ([21, 22]), show excellent resolution and accuracy that allows transmission of heavy ions up to an *m/z* of 40 000 ([23]).

Native MS has been applied to complex machines such as the ribosome [24, 25, 26, 27], viruses [28, 29, 30, 31, 32, 33] and membrane proteins [34, 35, 36, 37, 38, 39, 40]. These examples concern systems that are relatively well defined and are in some sense ‘static complexes’. However, native MS can also be applied to study dynamical molecular machines formed by multiple weak and mutually exclusive interactions [41, 42].

Here, we discuss, as a representative example, the application of native MS to the core machine for iron–sulfur (FeS) cluster biogenesis in *E. coli*. This specific example was not chosen by chance: one of the early native MS studies of a molecular assembly concerns precisely a FeS protein, *E. coli* biotin synthetase [43]. This protein was demonstrated to exist in solution as a mixture of monomer, dimer and tetramer and to be bound to a FeS cluster. We thus thought FeS clusters appropriate as a paradigm of the more general theme. Recently, native MS has been applied to the study of FeS binding proteins by an increasing number of groups who have confirmed its applicability [44, 45] and its great value to extract mechanistic details [46].

We analyse the advantages, problems and challenges proposed by native MS for the investigation of FeS cluster biogenesis based on our own experience in view of a wider perspective that goes beyond the specific application. We demonstrate that, while uniquely suited for the general purpose, further developments may be needed to make native MS fully applicable to the study of this challenging pathway.

## General overview of the FeS cluster biogenesis machine

**Fig. 1:**
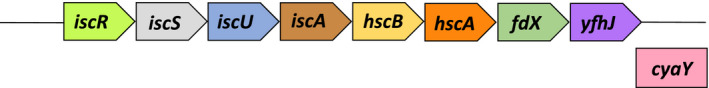
Schematic representation of the *isc* operon from *Escherichia coli.* It is composed of the genes encoding for the transcription regulator IscR, the desulfurase IscS, the scaffold protein IscU, IscA, a ferredoxin FdX, and two chaperone proteins HscB and HscA, and YfhJ. CyaY, the bacterial orthologue of frataxin, is encoded by a gene external to the *isc* operon.

Iron–sulfur cluster biogenesis is a representative example of a dynamic biological machine, which strongly relies on the formation and dissociation of different interactions. Also present in the inorganic world, FeS clusters are essential components of the cell since they are attached to proteins and have structural or redox roles [47]. In bacteria, the components are encoded in the *isc* operon and have high sequence homology to the eukaryotic ones (Fig. 1) [48]. The Isc machine comprises a number of essential proteins present among a promoter, a desulfurase IscS, a scaffold protein IscU, a putative alternative scaffold protein IscA, a ferredoxin that provides electrons FdX and two chaperone proteins HscB and HscA.

The IscS is a pyridoxal phosphate (PLP) binding enzyme that catalyses the conversion of L‐cysteine into L‐alanine and generates sulfur subsequently transferred to IscU [49] or to other proteins belonging to other metabolic pathways [50, 51]. It forms a complex with IscU, a small protein that transiently binds the cluster and passes it on to further acceptors. IscA is an alternative scaffold. Ferredoxin provides electrons for the FeS formation process. The two chaperones HscA and HscB are thought to assist cluster transfer [52]. Also involved in the machine is CyaY (frataxin in eukaryotes), an iron binding protein that is outside the operon. This protein has been widely studied because in humans it is associated with Friedreich’s ataxia [53]. It was firstly associated with the *isc* operon through interaction with HscB [54] and later on confirmed by pull‐down experiments [55, 56]. CyaY participates in FeS cluster formation as a regulator of the reaction speed [56, 57].

**Fig. 2:**
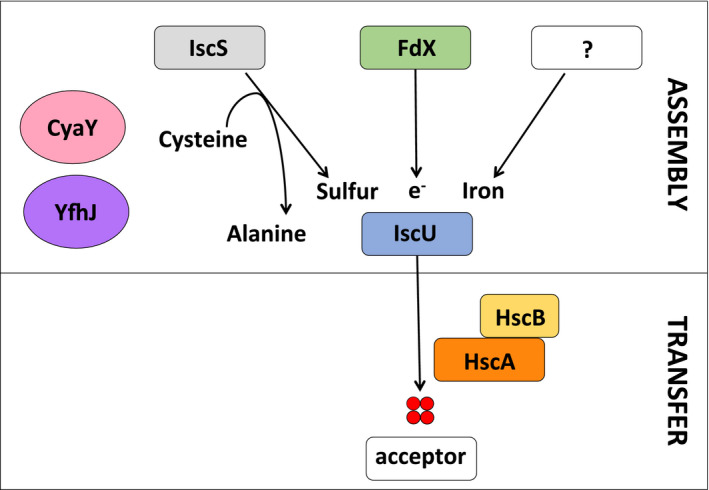
Schematic representation of the FeS cluster pathway of assembly. IscS converts cysteine into alanine and provides the sulfur to IscU. In the presence of the electron donor, FdX, and an iron donor, the FeS cluster forms on IscU. This process seems to be regulated by frataxin, CyaY, and probably YfhJ. The chaperone system of HscA and HscB assists the transfer of the FeS cluster from IscU to an apo‐acceptor.

Other ancillary proteins are selectively present in prokaryotes or in eukaryotes. Among them is YfhJ (also called IscX) that is specific for prokaryotes. This protein was suggested to be with frataxin, a regulator of the IscS activity depending on the iron concentration [58] (Fig. 2). Intriguingly, YfhJ, Fdx and CyaY compete for the same site on IscS [57, 59, 60, 61] and the binding affinity to IscS is modulated by the presence of iron cations.

## Strengths of using native MS for the study of the Isc system

**Fig. 3:**
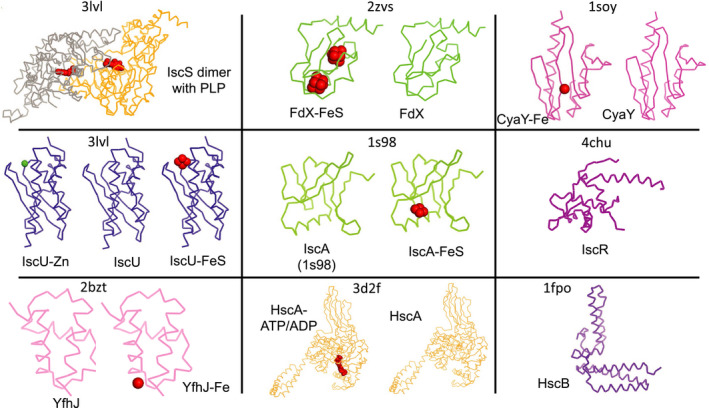
Summary of the main components of the *Isc* system in bacteria. Most of the Isc proteins involved in the FeS cluster biogenesis exist in different free and complexed forms bound to cofactors and/or ions that are essential for folding or function. When the structure of a specific form from *Escherichia coli* was not present in PDB, we modelled it from close orthologues to provide a visual impression of where the cofactor would be bound. The figure was produced using the pymol software (https://pymol.org/2/).

**Fig. 4:**
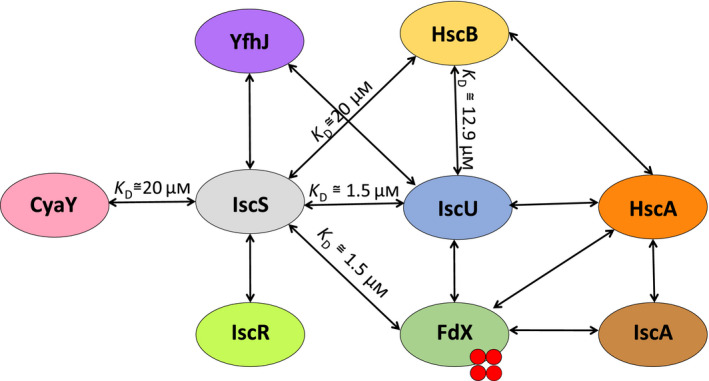
The Isc proteins map a complex network of interactions. Each arrow evidences an already‐certified direct molecular interaction between proteins that has been confirmed by more than one technique and reported by independent groups

**Fig. 5:**
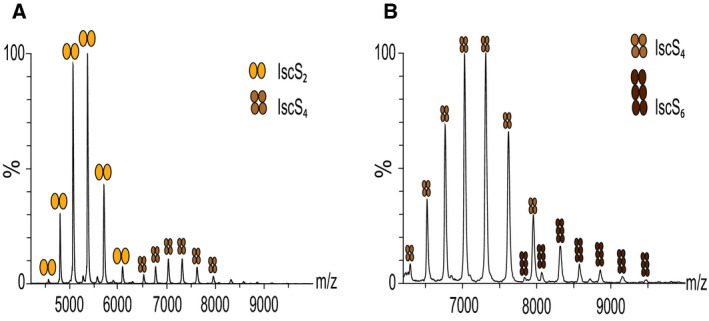
Control experiments to establish the feasibility and the sensitivity of the MS technique. (A) Native MS spectrum of 5 μM IscS in 250 mM ammonium acetate at pH 8. (B) Blow up of Figure 5A in the window 6500 to 10 000 m/z. Native MS revealed the presence of a minor group of peaks at higher m/z corresponding to the hexamer of IscS in addition to the signals corresponding to IscS dimer and tetramer.

Several are the challenges in the study of the FeS cluster core machine. While the Isc proteins and their interaction affinities are relatively well known, proving the existence and characterizing transient intermediates is a major challenge. Yet, this knowledge is indispensable for reconstructing the sequence of events occurring in cluster assembly and determining possible allosteric regulations. These multiple interactions are more complex also because many of the components can be in more than one state (e.g. metal‐bound/free, cluster‐bound/free, ATP/ADP‐bound/free and reduced/oxidized) (Fig. 3 and Table 1). The analysis of all the species formed thus requires a technique able to detect at the same time all the possible complexes in a range of molecular weights of 100‐200 KDa (Table **2**) and having different affinities ranging from 1 to 30 μm [62] (Fig. 4). It is here where native MS appears superior to most alternative techniques since it is able to retain protein structure, folding and interactions and detect the formation of transient complexes up to a molecular weight of MDa with a typical accuracy of a few Da. Only small amounts of samples are needed (5–10 μm), different ratios of the partners can be investigated, and no specific labels are necessary thus making the technique noninvasive. We also noticed that it is so sensitive to permit observation of minor species. We could, for instance, detect not only the IscS dimer, which is the dominant species in solution, but also the presence of an IscS tetramer and of a minor hexameric form (Fig. 5). The IscS tetramer could be detected also in size‐exclusion chromatography and by small‐angle scattering. Since the concentrations used in these studies are in the same order of magnitude observed for the same proteins in the cell [63], it is possible that these species may form under specific conditions, but their biological relevance is probably limited.

**Table 1:** Summary of the molecular weights of Isc proteins and their cofactors

Protein	MW (Da)	Metal/Cofactor	Metal/Cofactor MW (Da)
IscR	17 337	2Fe2S	176
IscS	45 089	PLP	247
IscU	13 849	Zn^2+^	65
		2Fe2S	176
IscA	11 556	2Fe2S	176
HscA	65 652	ADP	427
		ATP	507
HscB	20 138		
FdX	12 331	2Fe2S	176
		4Fe4S	352
YfhJ	7732	Fe^2+^/Fe^3+^	56
CyaY	12 231	Fe^2+^/Fe^3+^	56

**Table 2:** Isc complexes and molecular weights expected by mixing the desulfurase IscS with the scaffold protein IscU and frataxin CyaY, as example of the complexity of the system

Mixture	Complexes expected	MW (Da)
IscS	(IscS)_2_	90 178
IscS + IscU	(IscS)_2_(IscU)	104 027
	(IscS)_2_(IscU)_2_	117 876
IscS + IscU + CyaY	(IscS)_2_(IscU)(CyaY)	116 258
	(IscS)_2_(IscU)(CyaY)_2_	128 489
	(IscS)_2_(IscU)_2_(CyaY)	130 107
	(IscS)_2_(IscU)_2_(CyaY)_2_	142 338

## An essential control: retention of the enzymatic activity of the Isc system

**Fig. 6:**
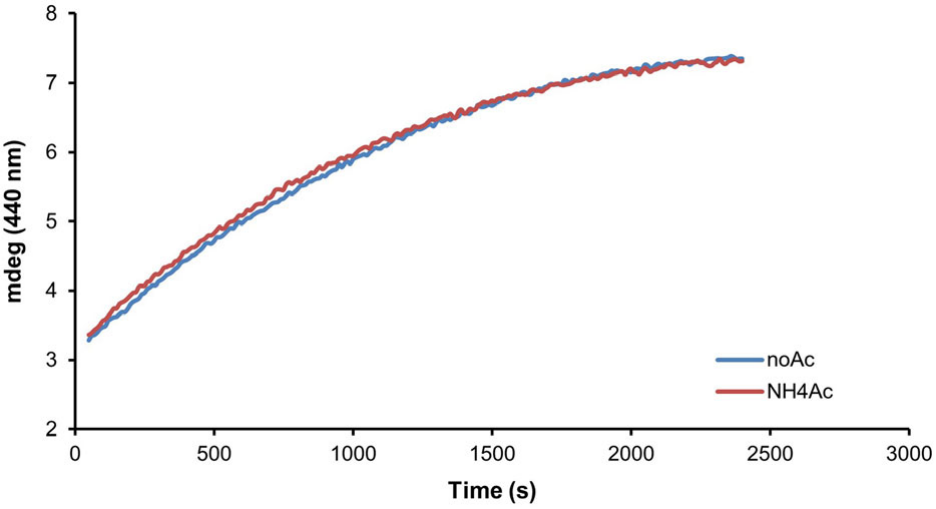
Kinetics of FeS cluster formation followed by absorbance spectra. Iron–sulfur clusters have a typical absorbance spectrum whose appearance demonstrates cluster formation. The enzymatic assay requires the presence of a source of sulfur (Cys), iron (iron ammonium sulfate), a desulfurase enzyme (IscS), a scaffold protein on which the cluster forms (IscU) and a reducing agent (DTT). The experiment was carried out using 50 μM IscU in the presence of 1 μM IscS, 250 μM Cys, 25 μM Fe(NH_4_)_2_(SO_4_)_2_ and 3 mM DTT in 20 mM Tris/HCl at pH 8, 150 mM NaCl at pH 8 (blue) or in 50 mm ammonium acetate at pH 8 (red).

Native MS experiments are usually performed using volatile buffers such as ammonium acetate [64, 65], ethylenediammonium diacetate or alkylammonium acetate buffers [66]. Unlike other types of ESI‐MS analysis, neither acidic conditions nor organic solvents are used. Typically, the buffer is exchanged immediately prior to native MS analysis [67]. The use of a volatile buffer might nevertheless represent a challenge for proteins that are marginally stable or not monodispersed as some of the components of the Isc system. Therefore, an essential control is to assess whether the enzymatic activity and cluster formation are affected by the experimental conditions dictated by the technique such as the presence and absence of ammonium acetate. This must be done under strict anaerobic conditions in a chamber kept under nitrogen atmosphere. The reaction can be followed by absorbance spectroscopy following absorbance variations at 458 nm or 406 nm and/or circular dichroism in the range 300‐600 nm as a function of time. Typical conditions for the reactions are 3 mM DTT, 50 μM IscU, 1 μM IscS and 25 μM Fe(NH_4_)_2_(SO_4_)_2_ preincubated for 30 min in 20 mM Tris/HCl at pH 8 and 150 mm NaCl [56]. The reaction is then initiated by adding 250 μM of the substrate L‐cysteine. The addition of CyaY (typically between 3 μM and 10 μM) inhibits the reaction [57]. In our hands, the system containing IscS, IscU and CyaY remains active in ammonium acetate provided that the buffer concentration does not exceed 50 mM (Fig. 6). Retention of the enzymatic activity also confirms that the experimental conditions do not alter protein structure.

## What can native MS report on the Isc system?

In this section, we shall discuss various aspects of the study of the FeS cluster biogenesis machine that native MS can assist.

### Using native MS to assess stoichiometry and symmetry

**Fig. 7:**
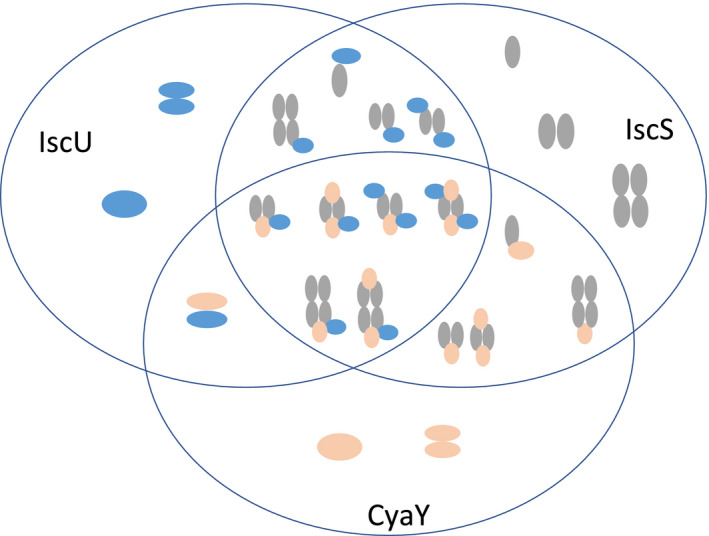
Potential artefacts of the technique. The MS spectrum shows species observed by native MS by mixing IscS 5 μM with IscU 5 μM and increasing concentration of CyaY up to 15 μM in 250 mM ammonium acetate at pH 8.

The IscS forms an obligate symmetric dimer composed of two identical 45 kDa subunits. At the interface between them, there is a cleft characterized by a positive charged surface due to the presence of three conserved arginines. This surface is a privileged site of interaction for protein negatively charged such as CyaY [57], YfhJ [58, 59] and Fdx [61]. Furthermore, it was shown that IscS is able to host HscB in the same pocket [68]. The same binding site is present on the symmetric site; thus, IscS can bind two molecules (of the same protein or two different proteins) at the same time. This opens the likely possibility of an allosteric regulation of the IscS desulfurase activity. In addition, each subunit of IscS is also able to bind IscU forming a complex with the stoichiometry of 1:1, meaning that the dimer binds two molecules of IscU. Native MS was applied to investigate whether IscS and other partners (e.g. CyaY) could form heterocomplexes in the presence or absence of IscU. In our studies, we typically injected a mixture of 5 μM IscS, 5 μM IscU and increasing molar ratios of CyaY ranging from 5 to 15 μM. We observed signals typical of the single components together with signals corresponding to noncovalent assemblies (Fig. 7). Data analyses indicated the presence of binary species, which could be identified as IscS_2_‐IscU, IscS_2_‐IscU_2_, IscS_2_‐CyaY, IscS_2_‐CyaY_2_ and CyaY‐IscU together with the heterocomplexes IscS_2_‐IscU‐CyaY, IscS_2_‐IscU_2_‐CyaY, IscS_2_‐IscU‐CyaY_2_ and IscS_2_‐IscU_2_‐CyaY_2_. This evidence also confirmed that binding of IscU to IscS does not compete with CyaY binding confirming previous studies [57].

### Validating unexpected interactions

**Fig. 8:**
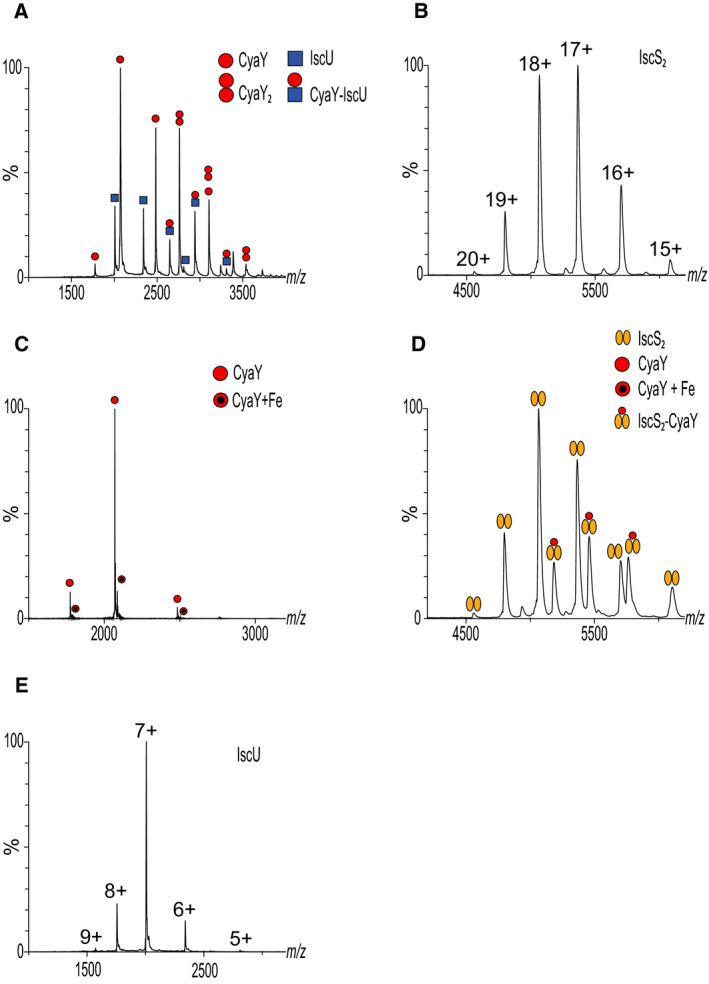
Exemplificative spectra. (A) Native MS spectrum of 5 μM IscU and 15 μM CyaY in 250 mM ammonium acetate at pH 8. We observed the signals belonging to the free proteins and to a new species corresponding to the complex CyaY‐IscU. (B) Native MS spectrum of IscS revealed that it is a dimer bound to two PLP molecules (with a total molecular weight of 91 073 Da). (C) In the presence of NH_4_Fe(SO_4_)_2_, native MS showed CyaY in both its forms free and bound Fe^3+^ with two NH^4+^. (D) By mixing CyaY and IscS in the presence of NH_4_Fe(SO_4_)_2_, a new species (CyaY‐IscS_2_ complex) is observed comparing to the spectrum of IscS alone. Unfortunately, the technique does not have enough resolution to determine whether CyaY binds IscS in the iron‐loaded or iron‐free form. (E) Native MS spectrum of 5 μM IscU in 250 mM ammonium acetate at pH 8 revealed only one distribution of peaks.

In our investigation of the Isc system by native MS, we also detected unexpected species such as complexes not previously described. For instance, we observed an IscU‐CyaY interaction (Fig. 8A): this has been repeatedly described between eukaryotic IscU and frataxin but not for the prokaryotic proteins [57, 69]. A key question is thus whether these interactions are biologically significant. In these doubtful cases, it is essential to use alternative techniques to validate the interactions independently. Approaches complementary to native MS may include NMR, ITC and/or cross‐linking. Each of these techniques has drawbacks, but consistent results obtained by different approaches may together validate the significance of newly disclosed interactions.

### Native MS preserves the binding of prosthetic groups

Some of the Isc components have constitutive binding partners such as prosthetic groups or ions. They are either essential for function (as it is PLP for IscS or ATP for HscA) or for fold stability (as it is zinc for IscU). It is widely accepted, for instance, that the reaction mechanism catalysed by IscS follows the rules proposed for the *A. vinelandii* homologue NIFS. In this mechanism, PLP would be essential for substrate activation through binding to the cysteine and forming an external aldimine base of Schiff prone to undergo a nucleophilic attack to liberate sulfur [70, 71]. Additionally, it was described that IscS remains enzymatically active only as long as it is bound to PLP [72]. Therefore, observing PLP on the desulfurase is essential to ensure correct folding of the enzyme and its activity.

IscU can bind a zinc metal ion in the same active site, which is responsible for hosting the cluster. This cation is thought to stabilize the folded form [73]. The presence of zinc does not alter the interaction with IscS and increases FeS cluster formation [74]. Eukaryotic frataxin and bacterial CyaY have been shown to form stable, although relatively weak, Fe^2+^ and Fe^3+^ complexes with a stoichiometry of up to six to seven iron ions [75, 76]. Also, YfhJ binds to iron in both its forms Fe^2+^ and Fe^3+^, through a negatively charged surface [77], and the surface responsible for the iron binding is the same which recognizes IscS.

We could prove that native MS preserves binding of all of these prosthetic groups to the respective protein: we clearly observed two molecules of PLP bound to the IscS dimer (Fig. 8B). We were also able to discriminate between iron‐loaded and iron‐free CyaY species (Fig. 8C). In other cases, especially when more than one protein was injected, many signals were present in a limited *m/z* range, making the data interpretation arduous. In these cases, it was not possible to determine whether loaded species or free ones were involved in binding (Fig. 8D).

### The challenge of working under anaerobic conditions

FeS clusters are a special type of prosthetic groups that may require specific experimental setting. They can be highly sensitive to oxygen [78] as oxygen species can convert them to unstable forms that rapidly decompose. This is particularly relevant for proteins of the core machine such as IscU that is able to bind FeS clusters but only in a weak and transient way. Under aerobic conditions, the cluster on IscU disassembles in a few minutes as it is testified by the absorbance spectrum or, more simply, by the disappearance of the dark red colour that characterizes the cluster‐loaded protein [72]. This is the reason why all the studies on the FeS cluster formation on this scaffold are carried out under strict anaerobic conditions. Unfortunately, the achievement of these conditions is often impractical or difficult with certain biophysical techniques. This is the case of the ion source in the native mass spectrometer that is not oxygen‐free. On the contrary, mass analysers and detectors are under vacuum. We could probably state that O_2_ exposure during the MS experiments may not be a problem because when the sample is infused into the ionization source, there is insufficient time for reaction to occur with any O_2_ present prior to ionization/introduction of low pressure. So far, we studied the interactions among Isc proteins in an aerobic environment and thus in the absence of the FeS clusters.

An alternative solution is to carry out the measurements on mutants that have higher affinity for the cluster. A mutant of IscU, for instance, that replaces a conserved aspartate to alanine (IscUD39A in *E. coli* IscU) loses the cluster slowly and can thus be used for the purpose [79]. This behaviour was explained by the elimination of a competition between the aspartate and a close‐by histidine: since both aspartate and histidine can chelate the cluster in a mutually exclusive fashion, mutation of one of the two residues leads to cluster stabilization [80]. In the past, we have indeed been able to observe a 2Fe2S cluster on the IscU mutant within hours from its anaerobic purification. In the future, we aim to build a dry box around the injection entrance of the spectrometer to be able to load the sample on the ESI needle and mount it on the source without exposure to oxygen. This will undoubtedly require some skill, but this step, even if delicate and cumbersome, will increase the potential applications of the instrument to oxygen‐sensitive proteins.

### Observing marginally stable protein conformations by MS

Proteins can assume multiple conformations depending on interactions, environmental conditions or intrinsic marginal stability [81]. Interconversion between conformations may be at the heart of their function. This is the case for at least one of the proteins involved in core FeS assembly, IscU. When produced by overexpression in *E. coli*, IscU is present in solution as an equilibrium between two distinct forms: a structured conformation (S‐state) and a partially dynamically disordered form (D‐state) [82]. The S‐state is stabilized by zinc, which binds in the same site that coordinates the cluster. When bound to IscS, IscU is in the S‐state [59, 83] but the D‐state was suggested to be relevant for cluster release. In the constitutive presence of zinc, a higher rate of cluster formation is observed, consistent with the presence of a more stable fold on which the cluster is assembled with the lower energetic cost [74]. In our hands and in our instrumental conditions, it was hard to distinguish between the two forms by native MS: we observed only one Gaussian distribution of signals (Fig. 8E).

More sophisticated experiments should be designed to probe for the presence of different conformations, such as coupling native MS with ion mobility (IM), which separates ions based on their differential mobility through a buffer gas [84, 85]. Another approach is to use hydrogen/deuterium exchange (HDX) MS [86, 87]. In this approach, folded conformations would have comparably fewer labile hydrogens readily accessible to the solvent and available for exchange than the unfolded conformation [88]. This generates differences between two populations, which could be assessed by MS.

## Conclusions

We have reviewed how native MS, a technique in use for several years but still relatively underexploited, can be adapted to the study of the FeS cluster biogenesis core machine. We discussed in detail the advantages and limitations of the technique applied to this specific system. We found that native MS offers several unique advantages over other techniques that range from the exquisite sensitivity, to the possibility to observe multiple states of the same protein within the instrumental resolution, to the possibility of looking at complex mixtures over a large mass range. This is of paramount importance for transient machines in which identification of the different complexes formed at different stages of the pathway matters, introducing a temporal dimension to the investigation.

In the context of a specific problem discussed here, a main current limitation remains certainly the difficulty to carry out the whole experiment anaerobically. Overcoming this aspect will require either *ad hoc* local solutions or a more general policy of the manufacturers, which could however not currently be justified given the limited numbers of users who might need these conditions. This might easily change in the future with the spreading of the technique. Another aspect that will require some consideration is the biological significance of minor species observed in some spectra, which will require comparison with measurements carried out by other techniques such as NMR or ITC. This step should anyway always be carried out as no technique can be trusted by its own. Despite these limitations, native MS undoubtedly constitutes an effective and powerful tool to be added to the already large range of biophysical techniques in place for the study of FeS cluster biogenesis. Its wider application to other similarly challenging machines may lead to a considerable step forward in our understanding of complex molecular processes in near future.

## Author contributions

RP carried out and wrote the first draft of the manuscript. EBE provided her expert knowledge in MS. AP finalized the manuscript.

## Conflict of interest

The authors declare no conflict of interest.

## References

[1] De Hoffmann E & Stroobant V (2007) Mass Spectrometry: Principles and Applications. Wiley.

[2] Thomson JJ . (1913) Rays of Positive Electricity and their Applications to Chemical Analysis. Longmans, Editor. Greens and Co.

[3] Fenn JB , Mann M , Meng C , Wong S & Whitehouse C (1989) Electrospray ionization for mass spectrometry of large biomolecules. Science 246, 64–71. 2675315 10.1126/science.2675315

[4] Chowdhury SK , Katta V & Chait BT (1990) Electrospray ionization mass spectrometric peptide mapping: a rapid, sensitive technique for protein structure analysis. Biochem Biophys Res Commun 167, 686–692. 2108669 10.1016/0006-291x(90)92080-j

[5] Tanaka K , Waki H , Ido Y , Akita S , Yoshida Y , Yoshida T & Matsuo T (1988) Protein and polymer analyses up to m/z 100 000 by laser ionization time‐of‐flight mass spectrometry. Rapid Commun Mass Spectrom 2, 151–153.

[6] Karas M & Hillenkamp F (1988) Laser desorption ionization of proteins with molecular masses exceeding 10,000 daltons. Anal Chem 60, 2299–2301. 3239801 10.1021/ac00171a028

[7] Last AM & Robinson CV (1999) Protein folding and interactions revealed by mass spectrometry. Curr Opin Chem Biol 3, 564–570. 10508674 10.1016/s1367-5931(99)00009-5

[8] Leney AC & Heck AJ (2017) Native Mass Spectrometry: What is in the Name? J Am Soc Mass Spectrom 28, 5–13. 10.1007/s13361-016-1545-3 PMC5174146 27909974

[9] Rostom AA & Robinson CV (1999) Disassembly of intact multiprotein complexes in the gas phase. Curr Opin Struct Biol 9, 135–141. 10047587 10.1016/s0959-440x(99)80018-9

[10] Tito MA , Tars K , Valegard K , Hajdu J & Robinson CV (2000) Electrospray time‐of‐flight mass spectrometry of the intact MS2 virus capsid. J Am Chem Soc 122, 3550–3551.

[11] Ilag LL , Videler H , McKay AR , Sobott F , Fucini P , Nierhaus KH & Robinson CV (2005) Heptameric (L12)6/L10 rather than canonical pentameric complexes are found by tandem MS of intact ribosomes from thermophilic bacteria. Proc Natl Acad Sci U S A 102, 8192–8197. 15923259 10.1073/pnas.0502193102 PMC1149426

[12] Boeri Erba E & Petosa C (2015) The emerging role of native mass spectrometry in characterizing the structure and dynamics of macromolecular complexes. Protein Sci 24, 1176–1192. 25676284 10.1002/pro.2661 PMC4534170

[13] Lossl P , van de Waterbeemd M & Heck AJ (2016) The diverse and expanding role of mass spectrometry in structural and molecular biology. EMBO J 35, 2634–2657. 27797822 10.15252/embj.201694818 PMC5167345

[14] Aquilina JA , Benesch JL , Bateman OA , Slingsby C , Robinson CV (2003) Polydispersity of a mammalian chaperone: mass spectrometry reveals the population of oligomers in alphaB‐crystallin. Proc Natl Acad Sci USA 100, 10611–10616. 12947045 10.1073/pnas.1932958100 PMC196852

[15] Patrick JW , Boone CD , Liu W , Conover GM , Liu Y , Cong X & Laganowsky A (2018) Allostery revealed within lipid binding events to membrane proteins. Proc Natl Acad Sci USA 115, 2976–2981. 29507234 10.1073/pnas.1719813115 PMC5866585

[16] Dyachenko A , Gruber R , Shimon L , Horovitz A & Sharon M (2013) Allosteric mechanisms can be distinguished using structural mass spectrometry. Proc Natl Acad Sci USA 110, 7235–7239. 23589876 10.1073/pnas.1302395110 PMC3645570

[17] Yee AW , Moulin M , Breteau N , Haertlein M , Mitchell EP , Cooper JB , Boeri Erba E & Forsyth VT (2016) Impact of Deuteration on the Assembly Kinetics of Transthyretin Monitored by Native Mass Spectrometry and Implications for Amyloidoses. Angew Chem Int Ed Engl 55, 9292–9296. 27311939 10.1002/anie.201602747 PMC5094506

[18] Yee AW , Aldeghi M , Blakeley MP , Ostermann A , Mas PJ , Moulin M , de Sanctis D , Bowler MW , Mueller‐Dieckmann C , Mitchell EP *et al*. (2019) A molecular mechanism for transthyretin amyloidogenesis. Nat Commun 10, 925. 30804345 10.1038/s41467-019-08609-z PMC6390107

[19] Sobott F , Hernández H , McCammon MG , Tito MA & Robinson CV (2002) A tandem mass spectrometer for improved transmission and analysis of large macromolecular assemblies. Anal Chem 74, 1402–1407. 11922310 10.1021/ac0110552

[20] van den Heuvel RH , van Duijn E , Mazon H , Synowsky SA , Lorenzen K , Versluis C , Brouns SJJ , Langridge D , van der Oost J , Hoyes J et al. (2006) Improving the performance of a quadrupole time‐of‐flight instrument for macromolecular mass spectrometry. Anal Chem 78, 7473–7483. 17073415 10.1021/ac061039a

[21] Makarov A (2000) Electrostatic axially harmonic orbital trapping: a high‐performance technique of mass analysis. Anal Chem 72, 1156–1162. 10740853 10.1021/ac991131p

[22] Makarov A , Denisov E , Kholomeev A , Balschun W , Lange O , Strupat K & Horning S (2006) Performance evaluation of a hybrid linear ion trap/orbitrap mass spectrometer. Anal Chem 78, 2113–2120. 16579588 10.1021/ac0518811

[23] Snijder J & Heck AJ (2014) Analytical approaches for size and mass analysis of large protein assemblies. Annu Rev Anal Chem (Palo Alto Calif) 7, 43–64. 25014341 10.1146/annurev-anchem-071213-020015

[24] Grela P , Krokowski D , Gordiyenko Y , Krowarsch D , Robinson CV , Otlewski J , Grankowski N & Tchórzewski M (2010) Biophysical properties of the eukaryotic ribosomal stalk. Biochemistry 49, 924–933. 20058904 10.1021/bi901811s

[25] Thoma C , Fraterman S , Gentzel M , Wilm M & Hentze MW (2008) Translation initiation by the c‐myc mRNA internal ribosome entry sequence and the poly(A) tail. RNA 14 (8), 1579–1589. 18556416 10.1261/rna.1043908 PMC2491467

[26] van de Waterbeemd M , Tamara S , Fort KL , Damoc E , Franc V , Bieri P , Itten M , Makarov A , Ban N & Heck AJR (2018) Dissecting ribosomal particles throughout the kingdoms of life using advanced hybrid mass spectrometry methods. Nat Commun 9, 2493. 29950687 10.1038/s41467-018-04853-x PMC6021402

[27] van de Waterbeemd M , Fort KL , Boll D , Reinhardt‐Szyba M , Routh A , Makarov A & Heck AJR (2017) High‐fidelity mass analysis unveils heterogeneity in intact ribosomal particles. Nat Methods 14, 283–286. 28114288 10.1038/nmeth.4147

[28] Weiss VU , Pogan R , Zoratto S , Bond KM , Boulanger P , Jarrold MF , Lyktey N , Pahl D , Puffler N , Schelhaas M *et al*. (2019) Virus‐like particle size and molecular weight/mass determination applying gas‐phase electrophoresis (native nES GEMMA). Anal Bioanal Chem 411, 5951–5962. 31280479 10.1007/s00216-019-01998-6 PMC6706367

[29] Veesler D , Khayat R , Krishnamurthy S , Snijder J , Huang RK , Heck AJR , Anand GS & Johnson JE (2014) Architecture of a dsDNA viral capsid in complex with its maturation protease. Structure 22, 230–237. 24361271 10.1016/j.str.2013.11.007 PMC3939775

[30] van de Waterbeemd M , Snijder J , Tsvetkova IB , Dragnea BG , Cornelissen JJ & Heck AJR (2016) Examining the Heterogeneous Genome Content of Multipartite Viruses BMV and CCMV by Native Mass Spectrometry. J Am Soc Mass Spectrom 27, 1000–1009. 26926442 10.1007/s13361-016-1348-6 PMC4869746

[31] Uetrecht C & Heck AJ (2011) Modern biomolecular mass spectrometry and its role in studying virus structure, dynamics, and assembly. Angew Chem Int Ed Engl 50, 8248–8262. 21793131 10.1002/anie.201008120 PMC7159578

[32] Kondylis P , Schlicksup CJ , Zlotnick A & Jacobson SC (2019) Analytical Techniques to Characterize the Structure, Properties, and Assembly of Virus Capsids. Anal Chem 91, 622–636. 30383361 10.1021/acs.analchem.8b04824 PMC6472978

[33] Bereszczak JZ , Barbu IM , Tan M , Xia M , Jiang X , van Duijn E & Heck AJR (2012) Structure, stability and dynamics of norovirus P domain derived protein complexes studied by native mass spectrometry. J Struct Biol 177, 273–282. 22266117 10.1016/j.jsb.2012.01.005

[34] Zhou M , Morgner N , Barrera NP , Politis A , Isaacson SC , Matak‐Vinkovic D , Murata T , Bernal RA , Stock D & Robinson CV (2011) Mass spectrometry of intact V‐type ATPases reveals bound lipids and the effects of nucleotide binding. Science 334, 380–385. 22021858 10.1126/science.1210148 PMC3927129

[35] Barrera NP , Di Bartolo N , Booth PJ & Robinson CV (2008) Micelles protect membrane complexes from solution to vacuum. Science 321, 243–246. 18556516 10.1126/science.1159292

[36] van Dyck JF , Konijnenberg A & Sobott F (2017) Native Mass Spectrometry for the Characterization of Structure and Interactions of Membrane Proteins. Methods Mol Biol 1635, 205–232. 28755371 10.1007/978-1-4939-7151-0_11

[37] Robinson CV , Rohacs T & Hansen SB (2019) Tools for Understanding Nanoscale Lipid Regulation of Ion Channels. Trends Biochem Sci 44, 795–806. 31060927 10.1016/j.tibs.2019.04.001 PMC6729126

[38] Laganowsky A , Reading E , Allison TM , Ulmschneider MB , Degiacomi MT , Baldwin AJ & Robinson CV (2014) Membrane proteins bind lipids selectively to modulate their structure and function. Nature 510, 172–175. 24899312 10.1038/nature13419 PMC4087533

[39] Gupta K , Li J , Liko I , Gault J , Bechara C , Wu D , Hopper JTS , Giles K , Benesch JLP & Robinson CV (2018) Identifying key membrane protein lipid interactions using mass spectrometry. Nat Protoc 13, 1106–1120. 29700483 10.1038/nprot.2018.014 PMC6049616

[40] Bechara C & Robinson CV (2015) Different modes of lipid binding to membrane proteins probed by mass spectrometry. J Am Chem Soc 137, 5240–5247. 25860341 10.1021/jacs.5b00420

[41] Snijder J , Rose RJ , Veesler D , Johnson JE , Heck AJR (2013) Studying 18 MDa virus assemblies with native mass spectrometry. Angew Chem Int Ed Engl 52, 4020–4023. 23450509 10.1002/anie.201210197 PMC3949431

[42] Shoemaker GK , van Duijn E , Crawford SE , Uetrecht C , Baclayon M , Roos WH , Wuite GJL , Estes MK , Prasad BVV & Heck AJR (2010) Norwalk virus assembly and stability monitored by mass spectrometry. Mol Cell Proteomics 9, 1742–1751. 20418222 10.1074/mcp.M900620-MCP200 PMC2938053

[43] Hernandez H , Hewitson KS , Roach P , Shaw NM , Baldwin JE , Robinson CV (2001) Observation of the iron‐sulfur cluster in *Escherichia coli* biotin synthase by nanoflow electrospray mass spectrometry. Anal Chem 73, 4154–4161. 11569804 10.1021/ac0102664

[44] Crack JC , Munnoch J , Dodd EL , Knowles F , Al Bassam MM , Kamali S , Holland AA , Cramer SP , Hamilton CJ , Johnson MK *et al*. (2015) NsrR from Streptomyces coelicolor is a nitric oxide‐sensing [4Fe‐4S] cluster protein with a specialized regulatory function. J Biol Chem 290, 12689–12704. 25771538 10.1074/jbc.M115.643072 PMC4432287

[45] Rao G , Pattenaude SA , Alwan K , Blackburn NJ , Britt RD , Rauchfuss TB (2019) The binuclear cluster of [FeFe] hydrogenase is formed with sulfur donated by cysteine of an [Fe(Cys)(CO)2(CN)] organometallic precursor. Proc Natl Acad Sci U S A 116, 20850–20855. 31570604 10.1073/pnas.1913324116 PMC6800375

[46] Parent A , Elduque X , Cornu D , Belot L , Le Caer J‐P , Grandas A , Toledano MB & D’Autréaux B (2015) Mammalian frataxin directly enhances sulfur transfer of NFS1 persulfide to both ISCU and free thiols. Nat Commun 6, 5686. 25597503 10.1038/ncomms6686

[47] Melber A & Winge DR (2018) Steps Toward Understanding Mitochondrial Fe/S Cluster Biogenesis. Methods Enzymol 599, 265–292. 29746243 10.1016/bs.mie.2017.09.004

[48] Roche B , Aussel L , Ezraty B , Mandin P , Py B & Barras F (2013) Iron/sulfur proteins biogenesis in prokaryotes: formation, regulation and diversity. Biochim Biophys Acta 1827, 455–469. 23298813 10.1016/j.bbabio.2012.12.010

[49] Urbina HD , Silberg JJ , Hoff KG & Vickery LE (2001) Transfer of sulfur from IscS to IscU during Fe/S cluster assembly. J Biol Chem 276, 44521–44526. 11577100 10.1074/jbc.M106907200

[50] Kambampati R & Lauhon CT (2000) Evidence for the transfer of sulfane sulfur from IscS to ThiI during the in vitro biosynthesis of 4‐thiouridine in *Escherichia coli* tRNA. J Biol Chem 275, 10727–10730. 10753862 10.1074/jbc.275.15.10727

[51] Lauhon CT & Kambampati R (2000) The iscS gene in *Escherichia coli* is required for the biosynthesis of 4‐thiouridine, thiamin, and NAD. J Biol Chem 275, 20096–20103. 10781607 10.1074/jbc.M002680200

[52] Puglisi R & Pastore A (2018) The role of chaperones in iron‐sulfur cluster biogenesis. FEBS Lett 592, 4011–4019. 30194723 10.1002/1873-3468.13245 PMC6506825

[53] Pastore A & Puccio H (2013) Frataxin: a protein in search for a function. J Neurochem 126 (Suppl 1), 43–52. 23859340 10.1111/jnc.12220

[54] Huynen MA , Snel B , Bork P , Gibson TJ (2001) The phylogenetic distribution of frataxin indicates a role in iron‐sulfur cluster protein assembly. Hum Mol Genet 10, 2463–2468. 11689493 10.1093/hmg/10.21.2463

[55] Gerber J , Muhlenhoff U & Lill R (2003) An interaction between frataxin and Isu1/Nfs1 that is crucial for Fe/S cluster synthesis on Isu1. EMBO Rep 4, 906–911. 12947415 10.1038/sj.embor.embor918 PMC1326356

[56] Adinolfi S , Iannuzzi C , Prischi F , Pastore C , Iametti S , Martin SR , Bonomi F & Pastore A (2009) Bacterial frataxin CyaY is the gatekeeper of iron‐sulfur cluster formation catalyzed by IscS. Nat Struct Mol Biol 16, 390–396. 19305405 10.1038/nsmb.1579

[57] Prischi F , Konarev PV , Iannuzzi C , Pastore C , Adinolfi S , Martin SR , Svergun DI & Pastore A (2010) Structural bases for the interaction of frataxin with the central components of iron‐sulphur cluster assembly. Nat Commun 1, 95. 20981023 10.1038/ncomms1097 PMC2982165

[58] Adinolfi S , Puglisi R , Crack JC , Iannuzzi C , Dal Piaz F , Konarev PV , Svergun DI , Martin S , Le Brun NE & Pastore A (2017) The Molecular Bases of the Dual Regulation of Bacterial Iron Sulfur Cluster Biogenesis by CyaY and IscX. Front Mol Biosci 4, 97. 29457004 10.3389/fmolb.2017.00097 PMC5801593

[59] Shi R , Proteau A , Villarroya M , Moukadiri I , Zhang L , Trempe J‐F , Matte A , Armengod ME & Cygler M (2010) Structural basis for Fe‐S cluster assembly and tRNA thiolation mediated by IscS protein‐protein interactions. PLoS Biol 8, e1000354. 20404999 10.1371/journal.pbio.1000354 PMC2854127

[60] Kim JH , Bothe JR , Frederick RO , Holder JC , Markley J (2014) Role of IscX in iron‐sulfur cluster biogenesis in *Escherichia coli* . J Am Chem Soc 136, 7933–7942. 24810328 10.1021/ja501260h PMC4063190

[61] Yan R , Konarev PV , Iannuzzi C , Adinolfi S , Roche B , Kelly G , Simon L , Martin SR , Py B , Barras F *et al*. (2013) Ferredoxin competes with bacterial frataxin in binding to the desulfurase IscS. J Biol Chem 288, 24777–24787. 23839945 10.1074/jbc.M113.480327 PMC3750173

[62] Prischi F & Pastore A (2017) Hybrid Methods in Iron‐Sulfur Cluster Biogenesis. Front Mol Biosci 4, 12. 28349052 10.3389/fmolb.2017.00012 PMC5346568

[63] Schmidt A , Kochanowski K , Vedelaar S , Ahrné E , Volkmer B , Callipo L , Knoops K , Bauer M , Aebersold R & Heinemann M (2016) The quantitative and condition‐dependent *Escherichia coli* proteome. Nat Biotechnol 34, 104–110. 26641532 10.1038/nbt.3418 PMC4888949

[64] Konermann L (2017) Addressing a Common Misconception: Ammonium Acetate as Neutral pH "Buffer" for Native Electrospray Mass Spectrometry. J Am Soc Mass Spectrom 28, 1827–1835. 28710594 10.1007/s13361-017-1739-3

[65] Hernandez H & Robinson CV (2007) Determining the stoichiometry and interactions of macromolecular assemblies from mass spectrometry. Nat Protoc 2, 715–726. 17406634 10.1038/nprot.2007.73

[66] Zhuang X , Gavriilidou AFM & Zenobi R (2017) Influence of Alkylammonium Acetate Buffers on Protein‐Ligand Noncovalent Interactions Using Native Mass Spectrometry. J Am Soc Mass Spectrom 28, 341–346. 27830529 10.1007/s13361-016-1526-6

[67] Boeri Erba E , Signor L , Oliva MF , Hans F , Petosa C (2018) Characterizing Intact Macromolecular Complexes Using Native Mass Spectrometry. Methods Mol Biol 1764, 133–151. 29605913 10.1007/978-1-4939-7759-8_9

[68] Puglisi R , Yan R , Adinolfi S & Pastore A (2016) A New Tessera into the Interactome of the isc Operon: A Novel Interaction between HscB and IscS. Front Mol Biosci 3, 48. 27730125 10.3389/fmolb.2016.00048 PMC5037179

[69] Layer G , Ollagnier‐de Choudens S , Sanakis Y & Fontecave M (2006) Iron‐sulfur cluster biosynthesis: characterization of *Escherichia coli* CYaY as an iron donor for the assembly of [2Fe‐2S] clusters in the scaffold IscU. J Biol Chem 281, 16256–16263. 16603772 10.1074/jbc.M513569200

[70] Zheng L , White RH , Cash VL & Dean DR (1994) Mechanism for the desulfurization of L‐cysteine catalyzed by the nifS gene product. Biochemistry 33, 4714–4720. 8161529 10.1021/bi00181a031

[71] Fujii T , Maeda M , Mihara H , Kurihara T , Esaki N , Hata Y (2000) Structure of a NifS homologue: X‐ray structure analysis of CsdB, an *Escherichia coli* counterpart of mammalian selenocysteine lyase. Biochemistry 39, 1263–1273. 10684605 10.1021/bi991732a

[72] Prischi F , Pastore C , Carroni M , Iannuzzi C , Adinolfi S , Temussi P & Pastore A (2010) Of the vulnerability of orphan complex proteins: the case study of the *E. coli* IscU and IscS proteins. Protein Expr Purif 73, 161–166. 20471481 10.1016/j.pep.2010.05.003

[73] Markley JL , Kim JH , Dai Z , Bothe JR , Cai K , Frederick RO & Tonelli M (2013) Metamorphic protein IscU alternates conformations in the course of its role as the scaffold protein for iron‐sulfur cluster biosynthesis and delivery. FEBS Lett 587, 1172–1179. 23333622 10.1016/j.febslet.2013.01.003 PMC3960074

[74] Iannuzzi C , Adrover M , Puglisi R , Yan R , Temussi PA & Pastore A (2014) The role of zinc in the stability of the marginally stable IscU scaffold protein. Protein Sci 23, 1208–1219. 24917298 10.1002/pro.2501 PMC4243993

[75] Yoon T & Cowan JA (2003) Iron‐sulfur cluster biosynthesis. Characterization of frataxin as an iron donor for assembly of [2Fe‐2S] clusters in ISU‐type proteins. J Am Chem Soc 125, 6078–6084. 12785837 10.1021/ja027967i

[76] Bou‐Abdallah F , Adinolfi S , Pastore A , Laue TM & Dennis Chasteen N (2004) Iron binding and oxidation kinetics in frataxin CyaY of *Escherichia coli* . J Mol Biol 341, 605–615. 15276847 10.1016/j.jmb.2004.05.072

[77] Pastore C *et al*. (2006) YfhJ, a molecular adaptor in iron‐sulfur cluster formation or a frataxin‐like protein? Structure 14, 857–867. 16698547 10.1016/j.str.2006.02.010

[78] Imlay JA (2003) Pathways of oxidative damage. Annu Rev Microbiol 57, 395–418. 14527285 10.1146/annurev.micro.57.030502.090938

[79] Foster MW *et al*. (2000) A mutant human IscU protein contains a stable [2Fe–2S]2 + center of possible functional significance. J Am Chem Soc 122, 6805–6806.

[80] Adrover M , Howes BD , Iannuzzi C , Smulevich G & Pastore A (2015) Anatomy of an iron‐sulfur cluster scaffold protein: Understanding the determinants of [2Fe‐2S] cluster stability on IscU. Biochim Biophys Acta 1853, 1448–1456. 25447544 10.1016/j.bbamcr.2014.10.023

[81] Pastore A , Martin SR & Temussi PA (2019) Generalized View of Protein Folding. In Medio Stat Virtus. J Am Chem Soc 141, 2194–2200. 30566837 10.1021/jacs.8b10779

[82] Kim JH , Füzéry AK , Tonelli M , Ta DT , Westler WM , Vickery LE & Markley JL (2009) Structure and dynamics of the iron‐sulfur cluster assembly scaffold protein IscU and its interaction with the cochaperone HscB. Biochemistry 48, 6062–6071. 19492851 10.1021/bi9002277 PMC2758247

[83] Yan R , Kelly G & Pastore A (2014) The scaffold protein IscU retains a structured conformation in the Fe‐S cluster assembly complex. ChemBioChem 15, 1682–1686. 25044349 10.1002/cbic.201402211

[84] Lanucara F , Holman SW , Gray CJ & Eyers CE (2014) The power of ion mobility‐mass spectrometry for structural characterization and the study of conformational dynamics. Nat Chem 6, 281–294. 24651194 10.1038/nchem.1889

[85] Ben‐Nissan G & Sharon M (2018) The application of ion‐mobility mass spectrometry for structure/function investigation of protein complexes. Curr Opin Chem Biol 42, 25–33. 29128665 10.1016/j.cbpa.2017.10.026 PMC5796646

[86] Mistarz UH & Rand KD (2018) Installation, validation, and application examples of two instrumental setups for gas‐phase HDX‐MS analysis of peptides and proteins. Methods 144, 113–124. 29753788 10.1016/j.ymeth.2018.05.002

[87] Kostyukevich Y , Acter T , Zherebker A , Ahmed A , Kim S & Nikolaev E (2018) Hydrogen/deuterium exchange in mass spectrometry. Mass Spectrom Rev 37, 811–853. 29603316 10.1002/mas.21565

[88] Katta V & Chait BT (1991) Conformational changes in proteins probed by hydrogen‐exchange electrospray‐ionization mass spectrometry. Rapid Commun Mass Spectrom 5, 214–217. 1666528 10.1002/rcm.1290050415

